# Balancing Iliac Crest Avoidance and Neural Safety in L5/S1 TELD: Quantitative Identification of a Candidate Balanced Trajectory Window Using AI‐Assisted 3D MRI Reconstruction

**DOI:** 10.1111/os.70433

**Published:** 2026-11-05

**Authors:** Yunfei Wang, Chengjie Huang, Qingqing He, Junjie Lu, Mengdi Li, Xiang Liu, Jingcheng Zhou, Ce Bian, Guiqian Zhang, Haotian Luo, Rui Zhang, Feng Liu, Xionggang Yang, Zhihai Su, Sheng Lu

**Affiliations:** ^1^ Department of Orthopedics The First People's Hospital of Yunnan Province and The Affiliated Hospital of Kunming University of Science and Technology, The Key Laboratory of Digital Orthopedics of Yunnan Province, Yunnan International Joint Laboratory of Intelligent Orthopedics, The Clinical Medicine Center of Spinal and Spinal Cord Disorders of Yunnan Province Kunming China; ^2^ Intelligent Orthopedics Medical Technology Research Centre of Kunming University of Science and Technology Kunming China; ^3^ Department of Spine Surgery The Fifth Affiliated Hospital of Sun Yat‐sen University Guangdong China; ^4^ Department of Nephrology The Second Affiliated Hospital of Chengdu Medical College (China National Nuclear Corporation 416 Hospital) Chengdu China; ^5^ Yunnan Key Laboratory of Optoelectronic Information Technology, College of Physics and Electronic Information Yunnan Normal University Kunming China; ^6^ Faculty of Information Engineering and Automation Kunming University of Science and Technology Kunming China; ^7^ Department of Orthopedic Trauma Qujing Central Hospital of Yunnan Province (Qujing Affiliated Hospital of Kunming Medical University) Qujing China; ^8^ Department of Spine Surgery The Second Affiliated Hospital of Guangzhou Medical University Guangzhou China

**Keywords:** AI‐assisted MRI segmentation, candidate balanced puncture angle, trajectory analysis, transforaminal endoscopic lumbar discectomy

## Abstract

**Objective:**

Transforaminal endoscopic lumbar discectomy (TELD) at L5/S1 is challenging due to the iliac crest barrier. Conventional planning relies on CT or 2D imaging, inherently lacking soft‐tissue contrast to visualize neural structures. This study aimed to define a quantitative candidate balanced trajectory zone using AI‐assisted 3D MRI reconstruction to simultaneously evaluate iliac crest obstruction and neural safety constraints.

**Methods:**

A deep learning model was trained on 50 thin‐slice lumbar MRIs to achieve precise segmentation and 3D reconstruction of neural, dural, and osseous structures. Thirty lumbar models were selected after image‐ and reconstruction‐quality screening for surgical puncture simulation. Using a 5° increment as the angular increment, 49 virtual working trajectories (coronal plane angles, CPA 0°–30° and cross‐sectional angles, CSA 0°–30°) were established for each bilateral intervertebral foramen, yielding a total of 2940 trajectories. Intersection volumes between each trajectory and the iliac crest, L5 nerve root, dural sac, and articular processes were quantitatively analyzed.

**Results:**

The model achieved an 86.4% mean Dice coefficient (73.2%–92.4%). Among 2940 trajectories, 1207 (41.1%) completely avoided the iliac crest. Spatial analysis revealed a trade‐off: increasing CSA reduced iliac and S1 superior articular process obstruction but increased L5 osseous removal, whereas steeper CPAs spared the L5 nerve root but risked the L5 inferior articular process. Quantitative analysis identified a candidate balanced trajectory window (CPA 20°–25°, CSA 25°–30°) that achieved a balance between iliac crest avoidance, neuro‐osseous preservation, and facet integrity.

**Conclusions:**

This candidate trajectory window (CPA 20°–25°/CSA 25°–30°) may provide a useful reference for balancing iliac crest avoidance with neuro‐osseous safety. These findings provide quantitative constraints for manual planning and for the future development of computer‐assisted trajectory‐planning systems.

## Graphical abstract

Using AI‐reconstructed 3D models, we defined an optimal trajectory zone (CPA 20°–25°, CSA 25°–30°) for L5/S1 TELD. This window safely bypasses the iliac crest while protecting neural structures, establishing critical geometric constraints for automated planning and surgical robotics.

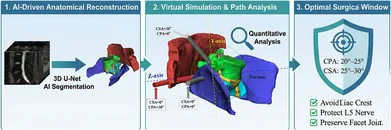

## 1 Introduction

Percutaneous endoscopic lumbar discectomy (PELD) particularly via the transforaminal approach has evolved into a standard minimally invasive procedure for lumbar disc herniation [1], offering significant advantages over traditional open surgery, such as reduced tissue trauma and faster recovery [2, 3]. Over decades, its indications have expanded to include spinal stenosis and recurrent protrusion [4]. However, the L5/S1 level remains the most technically challenging segment for transforaminal endoscopic lumbar discectomy (TELD) due to the unique anatomical “barrier” formed by the high iliac crest and narrow foramen [5, 6, 7, 8, 9]. Establishing a precise working channel is decisive for success, as suboptimal trajectories often necessitate repeated fluoroscopy [10, 11] and increase the risk of iatrogenic neural injury [4].

Currently, preoperative planning relies heavily on 2D imaging or CT‐based 3D reconstruction [11, 12, 13, 14]. While CT provides excellent bony detail, it inherently lacks the soft‐tissue contrast to visualize neural structures, leaving surgeons to estimate nerve root positions based on experience. Consequently, this reliance on empirical estimation significantly increases the risk of iatrogenic nerve injuries and a series of related postoperative complications [4, 15, 16, 17, 18]. Consequently, there is a need for quantitative preoperative evaluation frameworks integrating both osseous accessibility and neural safety considerations [14, 15].

Although MRI offers superior neural visualization [19], its application is limited by the time‐consuming nature of manual segmentation. In our previous work, we developed a deep learning‐based AI model capable of fully automated, rapid segmentation of complex lumbosacral structures—including L5/S1 vertebrae, the iliac crest, and critically, the L5 nerve roots and dural sac—from a three‐dimensional T2‐weighted SPACE (3D T2W‐SPACE) sequence [20]. This framework addresses the efficiency barrier of manual MRI segmentation and enables patient‐specific 3D reconstruction.

Previous studies have established the feasibility of AI‐assisted MRI segmentation and three‐dimensional reconstruction for lumbar endoscopic surgery [20]. Subsequent investigations have further explored the relationship between predefined trajectory parameters and structural interference within reconstructed lumbar models, primarily at the L4/L5 level [21]. However, for L5/S1 TELD, trajectory selection involves a balance among multiple competing factors, including iliac crest obstruction, neural safety, and facet preservation. A quantitative framework integrating these neuro‐osseous constraints within a unified MRI‐derived three‐dimensional environment remains insufficiently explored.

Building on these AI‐assisted reconstructions, we simulated thousands of virtual working channels to quantitatively analyze the spatial relationships between surgical trajectories and neuro‐osseous structures. The primary objective was to define a candidate balanced trajectory zone for L5/S1 TELD. These findings aim to guide manual planning and provide quantitative anatomical constraints for future computer‐assisted planning software.

## 2 Materials and Methods

### 2.1 Ethics and Registration

This study was approved by the Institutional Review Board (Approval No. 2020 K05‐1) and conducted in accordance with the Declaration of Helsinki and STROCSS 2021 guidelines [22]. Written informed consent was obtained from all participants (NCT: ChiCTR2100052883).

### 2.2 Patient Characteristics

This study retrospectively enrolled 50 patients diagnosed with L5/S1 lumbar disc herniation between March and July 2020. The inclusion criteria were: (1) confirmed diagnosis of L5/S1 herniation; (2) age ≥ 18 years; and (3) availability of high‐quality lumbar MRI. Exclusion criteria strictly ruled out patients with prior spinal surgery, spinal deformities (e.g., scoliosis, spondylolisthesis), infections, tumors, or severe motion artifacts. The final cohort included patients aged 23–84 years (mean: 43.7) with a BMI range of 17.72–33.03 kg/m^2^. Imaging was performed using a Siemens 3.0 T MRI system (Magnetom Verio, Erlangen, Germany). To ensure isotropic resolution suitable for 3D reconstruction, we utilized a 3D T2W‐SPACE sequence covering the L5 superior endplate to the S1 inferior endplate. Detailed acquisition parameters were: TR/TE = 2800.0/189.0 ms, FOV = 14.0 cm, flip angle = 45°, matrix = 320 × 320, and a voxel size of 0.8 × 0.8 × 0.8 mm^3^. Because the 3D T2W‐SPACE sequence requires a relatively long acquisition time and is susceptible to motion‐related degradation, patients with severe motion artifacts were excluded before model reconstruction.

### 2.3 Ground Truth Generation and AI Model Development

The dataset of 50 MRI volumes was utilized to train a deep learning segmentation model based on a 3D U‐Net architecture [20] (Figure 1). To establish the ground truth, three experienced spine specialists manually segmented seven anatomical classes using Mimics software (Materialise, Leuven, Belgium): L5 and S1 vertebrae, the L5/S1 intervertebral disc, the ilium, L5 nerve roots, dural sac, and skin. A consensus voting mechanism was employed to resolve discrepancies in boundary delineation. Following training and validation, the model generated voxel‐based 3D masks. The trained model was first applied to reconstruct all 50 MRI‐based lumbosacral models. For the present trajectory analysis, we selected the 30 reconstructions with the best overall mask continuity, anatomical integrity, and surface quality. For these 30 models, no extensive slice‐by‐slice manual mask editing, manual signal erasure, or defect filling was performed to create artificially regular masks. Post‐processing was limited to simple visualization‐oriented operations, such as minor sharpness or surface‐display adjustment, to improve model appearance and facilitate intuitive observation; these operations did not alter the anatomical masks used for quantitative intersection analysis. To document the provenance of the imaging data and the complete reconstruction workflow, we have provided an unedited continuous screen‐recording video showing the process from raw MRI DICOM import through deep learning segmentation, mask generation, and three‐dimensional reconstruction (Video S1).

**FIGURE 1:**
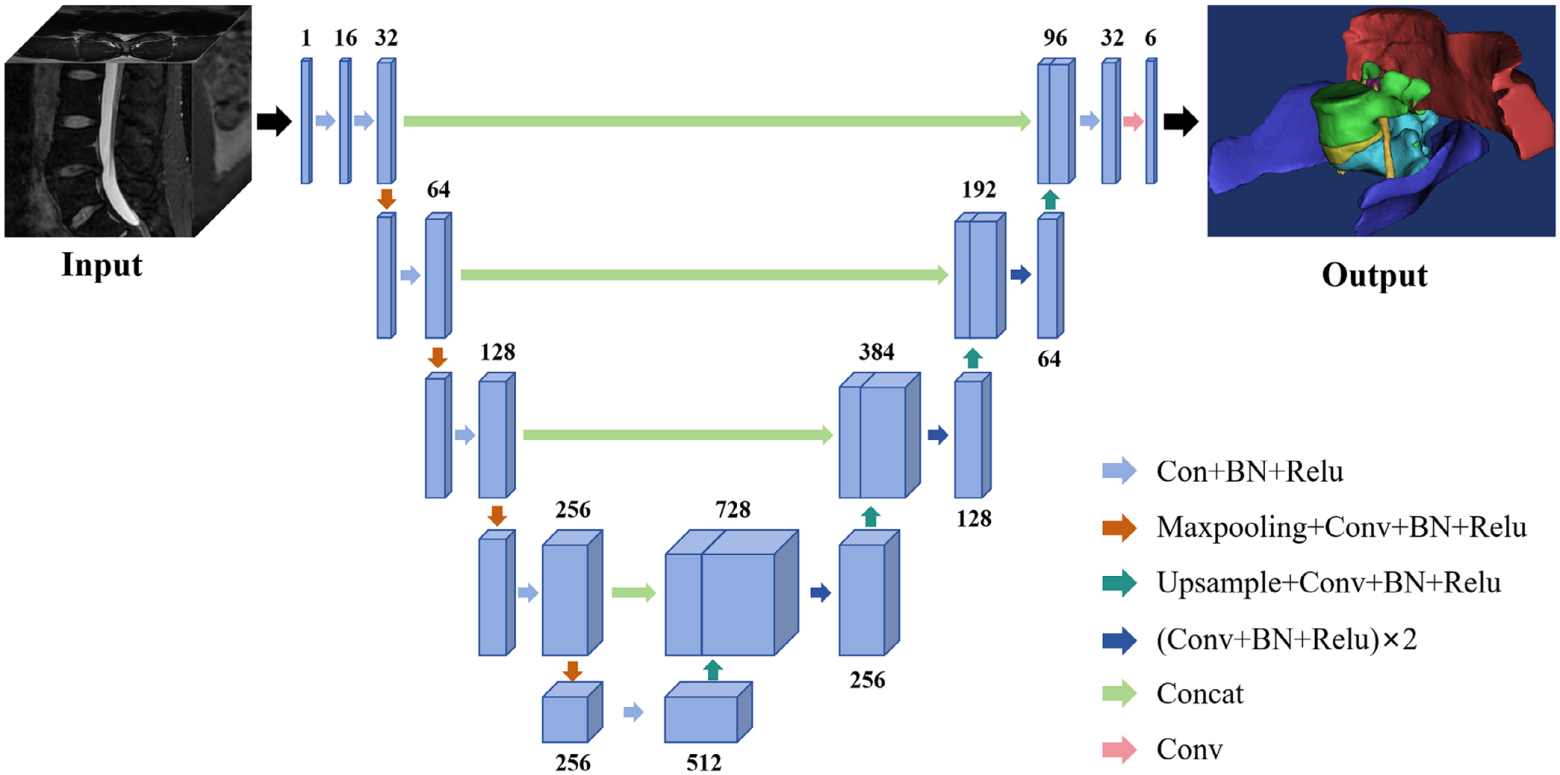
Schematic illustration of the 3D U‐Net based deep learning architecture for the automated segmentation of lumbosacral structures from MRI data.

### 2.4 Virtual Trajectory Simulation and Quantitative Analysis

Automated voxel‐based masks were reconstructed into 3D polygonal surface meshes in Mimics 19.0. The surgical target point was standardized as the projection of the medial border of the bilateral facet joints onto the midline of the intervertebral disc space. A virtual working channel—modeled as a solid geometric cylinder (8 mm diameter, 150 mm length)—was generated to simulate a rigid TELD cannula.

Within the 3‐Matic environment, a local coordinate system was established with its origin centered on the target point. The cylindrical channel was initially aligned at a neutral 0° trajectory and subsequently subjected to incremental angular rotations along the coronal plane angle (CPA, *Z*‐axis) and cross‐sectional angle (CSA, *Y*‐axis) ranging from 0° to 30° at 5° intervals (Figure 2A). As both the CPA and CSA were adjusted across seven discrete angles (0°, 5°, 10°, 15°, 20°, 25°, and 30°), a 7 × 7 combination matrix was formed, yielding 49 unique virtual trajectories per intervertebral foramen. With the simulations conducted bilaterally (two foramina per model) on all 30 patient‐specific 3D models, a comprehensive dataset of 2940 distinct surgical trajectories (30 models × 2 foramina/model × 49 trajectories/foramen) was systematically generated. Notably, all trajectories across the full 7 × 7 angular matrix—including those expected to intersect the iliac crest—were deliberately included in the initial simulation to avoid selection bias and to permit a complete, quantitative characterization of the spatial transition from high‐obstruction to low‐obstruction corridors. This comprehensive mapping strategy enables the identification of precise angular thresholds where iliac crest interference becomes quantitatively reduced, thereby defining the boundary conditions of the candidate balanced trajectory window (Figure 2A).

**FIGURE 2:**
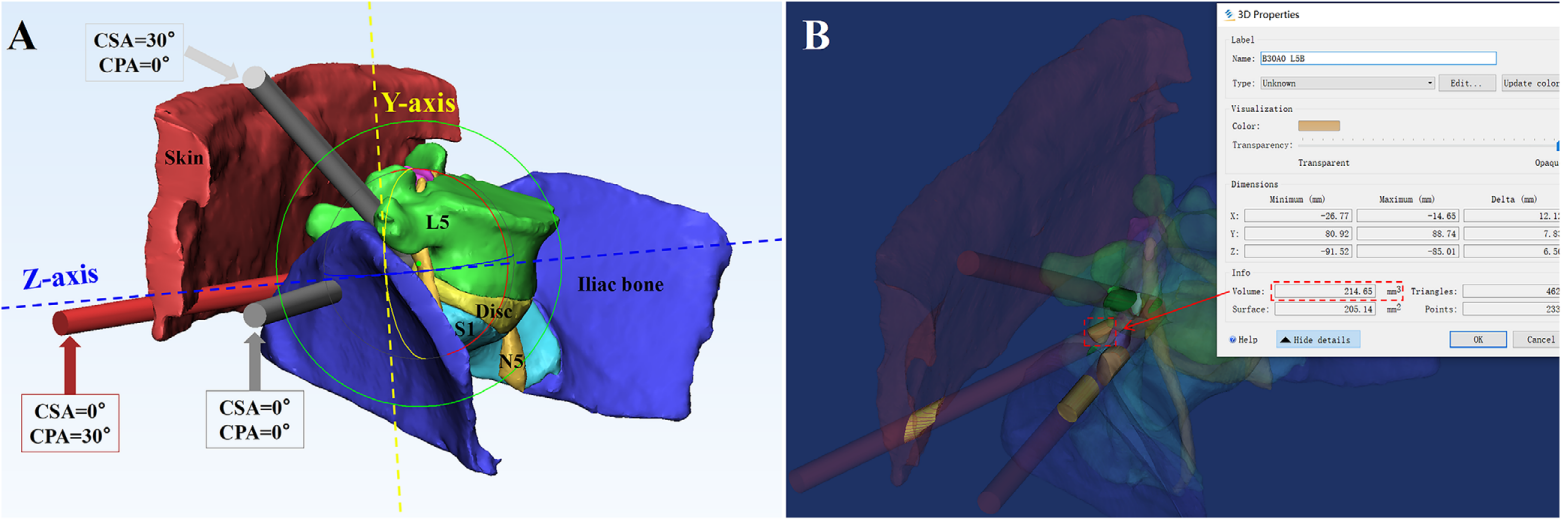
Definition of virtual trajectory parameters and quantitative intersection analysis. (A) Establishment of the local coordinate system on the 3D lumbosacral model. The *Z*‐axis defines the coronal plane angle (CPA), and the *Y*‐axis defines the cross‐sectional angle (CSA). Three representative working channels are depicted at distinct configurations: Neutral (CSA 0°/CPA 0°), pure coronal tilt (CSA 0°/CPA 30°), and pure cross‐sectional tilt (CSA 30°/CPA 0°). (B) Visualization of the volumetric intersection calculation. The arrow highlights a quantified intersection volume of 214.65 mm^3^ occurring between the virtual channel (CSA 0°/CPA 30°) and the L5 inferior articular process.

To quantify surgical invasiveness and obstruction, an automated volumetric intersection analysis was performed using the built‐in tools of Mimics to calculate the intersection volume (spatial collision) between the solid cylinder and the adjacent anatomical structures. Specifically, the volumetric intersections were categorized into critical neuro‐osseous constraints (L5 nerve root, L5 inferior articular process‐IAP, S1 superior articular process [SAP], and iliac crest) and atypical osseous boundaries (vertebral bodies and pedicles). All intersection volumes were automatically computed to assess the extent of spatial overlap with neural and osseous structures (Figure 2B). Because validated clinical thresholds for acceptable intersection volumes are not currently available, and because different structures carry different clinical implications, we did not apply a fixed cutoff or formal weighting function. Instead, the final trajectory window was defined descriptively as a candidate balanced zone that showed low iliac crest and neural intersection while avoiding excessive facet‐joint violation across the simulated angle grid.

### 2.5 Statistical Analysis

Statistical processing was performed using IBM SPSS Statistics Version 26. The segmentation performance was evaluated via Dice coefficient, accuracy, and recall. A bivariate Spearman's rank correlation analysis was conducted to determine the association between trajectory angles and intersection volumes, with statistical significance defined as *p* < 0.05.

## 3 Results

### 3.1 Performance of the Automated Segmentation Model

The 3D U‐Net based deep learning model achieved an overall mean Dice coefficient of 0.864, an accuracy of 0.875, and a recall of 0.866 for lumbosacral structures (Table 1). For osseous structures, the Dice coefficients for vertebrae were > 0.92. For soft tissues, the model yielded Dice coefficients of 0.885 for the dural sac and 0.732 for the L5 nerve roots.

**TABLE 1:** Performance of the automatic MRI segmentation model.

Performance	Multiclass spinal structures							
	L5	Disc	S1	Iliac crest	L5 nerve root	Dural sac	Skin	Mean
Dice	0.921	0.885	0.924	0.886	0.732	0.885	0.816	0.864
Accuracy	0.939	0.879	0.928	0.897	0.766	0.893	0.821	0.875
Recall	0.906	0.907	0.923	0.883	0.716	0.884	0.845	0.866

*Note:* Data are means of fivefold cross‐validation scores.

Abbreviations: L5 = fifth lumbar vertebra bone, S1 = first sacral vertebra bone.

### 3.2 Intersection Volumes Assessment Results

#### 3.2.1 Intersection Analysis: Iliac Crest

Among the 2940 simulated trajectories, 1207 (41.1%) showed no intersection (0 mm^3^) with the iliac crest. Statistical analysis indicated a significant negative correlation between the trajectory angles and iliac intersection volumes (Table 2). The intersection volume decreased as the CSA increased (*r* = −0.592, *p* < 0.001). Increasing the CPA resulted in an initial increase followed by a decrease in intersection volume (r = −0.145, *p* < 0.001) (Figure 3A). The lowest mean iliac‐crest intersection volume across the 49 angular configurations was 14.81 mm^3^ at CPA 30°/CSA 30°.

**TABLE 2:** Spearman's rank correlation analysis of intersection volumes between virtual working channels and lumbar structures at different angles.

		L5 IAP	L5 R	L5 All	S1 SAP	S1 R	S1 All	L5 nerve root	Dural sac	Iliac crest
CSA	*r*	−0.195	0.650	0.631	−0.391	−0.563	−0.713	0.150	0.179	−0.592
	*p*	< 0.001	< 0.001	< 0.001	< 0.001	< 0.001	< 0.001	< 0.001	< 0.001	< 0.001
CPA	*r*	0.546	−0.384	−0.009	0.129	−0.225	−0.032	−0.550	0.333	−0.145
	*p*	< 0.001	< 0.001	0.637	< 0.001	< 0.001	0.083	< 0.001	< 0.001	< 0.001

Abbreviations: CPA, coronal plane angle; CSA, cross‐section angle; L5 All, all structures of the fifth lumbar vertebra bone; L5 IAP, inferior articular process of the fifth lumbar vertebra bone; L5 R, the atypical structures of the fifth lumbar vertebra bone (including the vertebral body, pedicles, and transverse processes); *r*, Spearman correlation coefficient; S1 All, all structures of the first sacral vertebra bone; S1 R, the atypical structures of the first sacral vertebra bone (including the vertebral body, pedicles, and transverse processes); S1 SAP, superior articular process of the first sacral vertebra bone.

**FIGURE 3:**
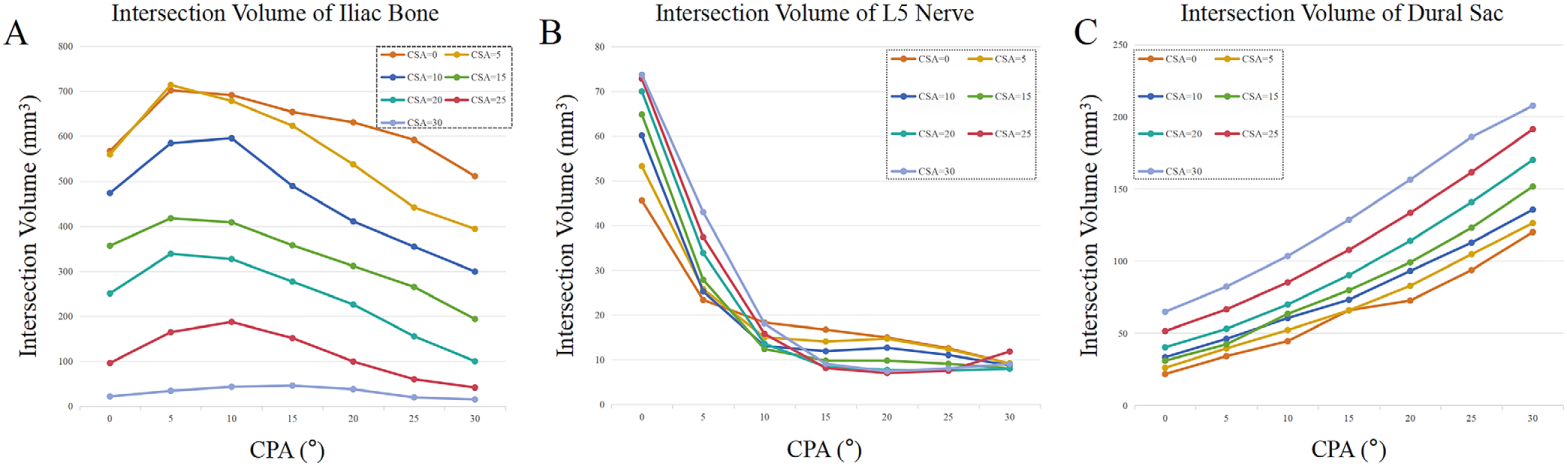
Average intersection volumes between virtual working channels and critical neuro‐osseous barriers across varying cross‐sectional angles (CSA) and coronal plane angles (CPA): (A) Volume with the iliac crest. (B) Volume with the L5 nerve root. (C) Volume with the dural sac.

#### 3.2.2 Intersection Analysis: Neural Structures (L5 Nerve Root and Dural Sac)

As shown in Table 2, a moderate negative correlation was observed between the CPA and L5 nerve root intersection volume (*r* = −0.550, *p* < 0.001), indicating that steeper CPA was associated with a lower simulated L5 nerve intersection volume. Conversely, CSA showed a weak positive correlation (*r* = 0.150, *p* < 0.001). The L5 nerve root intersection volume generally decreased with increasing CPA and remained relatively low across many higher‐angle configurations. However, this pattern was not uniform across all combinations; for example, the CPA 30°/CSA 25° configuration showed a mean intersection volume of 11.81 mm^3^ (Figure 3B). Detailed intersection volumes are provided in Table 3.

**TABLE 3:** Mean intersection volumes between virtual working channels and the L5 inferior articular process, L5 nerve root, S1 superior articular process, and iliac crest across different angular configurations.

Different angle and intersection volumes (mm^3^)	CPA 0°	CPA 5°	CPA 10°	CPA 15°	CPA 20°	CPA 25°	CPA 30°
CSA 0°							
L5 IAP	0.19	0.06	1.24	7.14	28.17	91.33	182.56
L5 nerve root	45.62	23.37	18.30	16.72	14.96	12.54	9.16
S1 SAP	95.02	224.63	336.07	336.15	295.61	244.93	190.89
Iliac crest	567.48	702.34	691.73	654.17	631.15	592.09	511.44
CSA 5°							
L5 IAP	0.70	0.23	0.53	10.63	46.05	127.56	221.40
L5 nerve root	53.27	25.79	15.01	14.06	14.69	12.32	9.19
S1 SAP	50.88	149.99	216.10	267.60	301.20	261.80	167.41
Iliac crest	559.72	714.41	678.55	623.55	537.42	442.02	393.82
CSA 10°							
L5 IAP	1.20	0.52	1.56	10.46	57.35	157.57	270.91
L5 nerve root	60.15	25.27	13.05	11.88	12.65	11.03	8.74
S1 SAP	30.86	103.06	178.66	244.87	254.20	200.98	127.63
Iliac crest	473.79	584.76	595.79	489.55	410.68	354.49	299.12
CSA 15°							
L5 IAP	3.17	3.22	3.70	9.30	56.20	146.36	264.56
L5 nerve root	64.88	27.89	12.36	9.80	9.81	9.10	8.05
S1 SAP	25.08	65.90	137.70	166.10	171.67	128.70	81.46
Iliac crest	356.55	417.71	408.59	357.65	311.36	264.77	193.45
CSA 20°							
L5 IAP	13.87	13.14	10.49	17.77	46.53	109.81	203.94
L5 nerve root	70.02	33.86	13.57	8.41	7.71	7.58	7.98
S1 SAP	19.93	42.64	73.29	95.13	92.45	71.26	50.68
Iliac crest	250.37	338.62	326.82	276.71	225.94	155.19	99.72
CSA 25°							
L5 IAP	19.83	22.87	19.18	26.78	33.47	74.74	102.81
L5 nerve root	72.88	37.41	15.81	8.13	7.01	7.52	11.81
S1 SAP	20.10	26.55	37.47	45.46	45.29	39.42	32.31
Iliac crest	95.66	164.41	186.97	151.16	99.07	59.44	40.99
CSA 30°							
L5 IAP	20.80	23.44	23.53	29.56	24.65	40.79	71.67
L5 nerve root	73.73	43.04	18.06	9.07	7.41	8.04	9.06
S1 SAP	21.42	20.74	21.97	24.41	48.74	23.26	22.46
Iliac crest	21.45	33.78	42.66	45.18	37.34	19.29	14.81

*Note:* Data are means of intersection volume (mm^3^).

Abbreviations: CPA, coronal plane angle; CSA, cross section angle; L5 IAP, inferior articular process of fifth lumbar vertebra bone; S1 SAP, superior articular process of first sacral vertebra bone.

In contrast, the dural sac intersection volume demonstrated a positive correlation with both CPA (*r* = 0.333, *p* < 0.001) and CSA (*r* = 0.179, *p* < 0.001) (Table 2), suggesting that greater angulation was associated with increased simulated intersection with the dural sac (Figure 3C). Detailed intersection volumes are provided in Table S1.

#### 3.2.3 Intersection Analysis: L5 Vertebra and IAP

The total intersection volume between the virtual working channel and the L5 vertebrae is shown in Figure 4A. Intersection volumes generally decreased with increasing CPA (*r* = −0.009, *p* = 0.637) but increased with higher CSA (*r* = 0.631, *p* < 0.001) (Table 2). For the L5 atypical structures (including the vertebral body, pedicle, and transverse process), the intersection volume demonstrated a moderate positive correlation with CSA (*r* = 0.650, *p* < 0.001), indicating that larger CSAs lead to greater bone removal in these areas (Figure 4B). Conversely, a negative correlation was observed with CPA (*r* = −0.384, *p* < 0.001) (Table 2).

**FIGURE 4:**
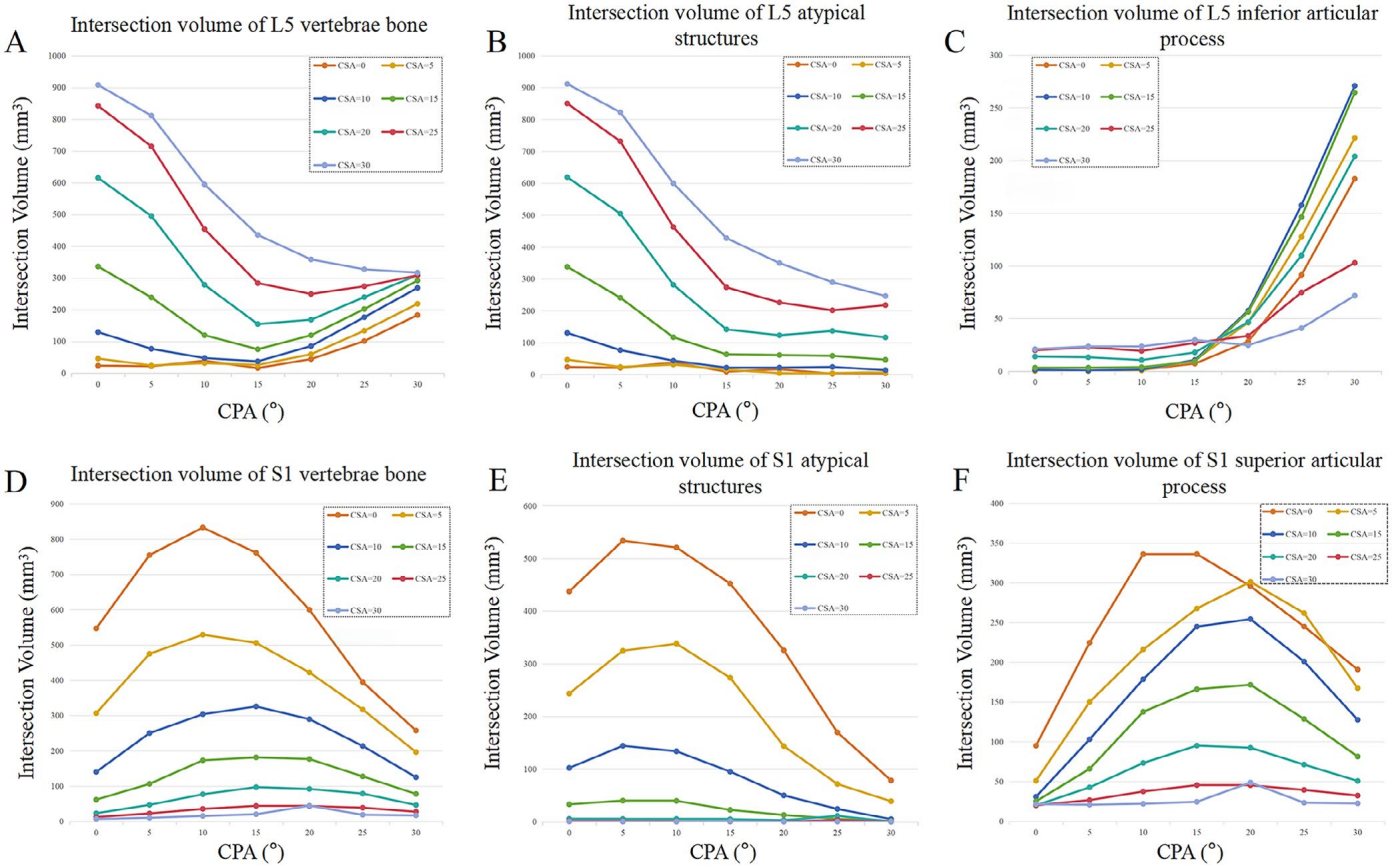
Average intersection volumes between virtual working channels and L5/S1 osseous structures across varying cross‐sectional angles (CSA) and coronal plane angles (CPA). (A) Total intersection volume with the entire L5 vertebra. (B) Volume with L5 atypical structures (vertebral body, pedicle, and transverse process). (C) Volume with the L5 inferior articular process. (D) Total intersection volume with the entire S1 vertebra. (E) Volume with S1 atypical structures. (F) Volume with the S1 superior articular process.

However, the L5 IAP—a critical stabilizing structure—exhibited an opposing trend: its intersection volume decreased as CSA increased (*r* = −0.195, *p* < 0.001) but increased with higher CPA (*r* = 0.546, *p* < 0.001) (Table 2). Thus, a larger CSA is beneficial for preserving the facet joint but may increase the risk of collision with the transverse process or pedicle (Figure 4C). Detailed data regarding the intersection volumes between the working channels at varying angles and the L5 vertebra are summarized in Tables 3 and S1.

#### 3.2.4 Intersection Analysis: S1 Vertebra and SAP

Figure 4D illustrates the trends of the total intersection volume between the virtual working channel and the S1 vertebral body across varying angles. The intersection volumes for S1 structures generally decreased as the CSA increased (*r* = −0.713, *p* < 0.001). In contrast to L5, the intersection volumes for S1 atypical structures showed a consistent negative correlation with CSA (*r* = −0.563, *p* < 0.001). Regarding the CPA, the volume initially increased but subsequently decreased as the angle exceeded 20°, showing an overall weak negative correlation (*r* = −0.225, *p* < 0.001) (Table 2 and Figure 4E).

Similarly, the S1 SAP volume decreased significantly with increasing CSA (*r* = −0.391, *p* < 0.001) (Table 2). Therefore, increasing the CSA is generally protective for both the S1 facet joint and atypical bony structures (Figure 4F). Detailed data regarding the intersection volumes are provided in Tables 3 and S1.

## 4 Discussion

### 4.1 AI‐Assisted Reconstruction

In this study, we established a two‐stage framework combining AI‐assisted anatomical reconstruction and rule‐based geometric trajectory analysis to quantitatively characterize the complex spatial relationship between the TELD working channel and neuro‐osseous structures at the L5/S1 level. In the first stage, a 3D U‐Net deep learning model enabled automated reconstruction of seven lumbosacral structures from high‐resolution MRI data, overcoming the major efficiency limitation associated with manual MRI segmentation. This process generated patient‐specific three‐dimensional anatomical models containing both osseous structures and critical soft tissues, including the L5 nerve roots and dural sac.

Previous studies have demonstrated the feasibility of AI‐assisted MRI segmentation and three‐dimensional reconstruction for lumbar endoscopic surgery. Subsequent investigations further explored trajectory‐related anatomical relationships based on reconstructed models [20, 21]. However, these studies primarily focused on anatomical reconstruction or isolated trajectory–structure interactions. In contrast, the present study extends this concept by using MRI‐derived models to quantitatively evaluate the competing anatomical constraints involved in L5/S1 TELD trajectory selection. Rather than simply identifying a geometrically accessible pathway, our framework analyzes the balance among iliac crest obstruction, neural structure preservation, and facet‐related osseous limitations.

Importantly, the AI component in this study served as an anatomical reconstruction tool, while the subsequent trajectory assessment was performed using predefined geometric simulations. This distinction allows the current framework to provide quantitative anatomical boundary conditions without assuming that AI itself determines the surgical trajectory. Therefore, this study represents a further step from three‐dimensional anatomical visualization toward quantitative evaluation of trajectory assessment.

### 4.2 Neuro‐Osseous Trade‐Offs in Trajectory Selection

L5/S1 TELD remains technically challenging because the working corridor is simultaneously restricted by the high iliac crest, narrow foramen, and adjacent neural and facet structures [23]. Traditional CT‐based planning provides detailed information regarding bony accessibility but cannot directly quantify neural interference [24, 25]. By incorporating MRI‐derived visualization of neural structures, our study provides a more comprehensive assessment of the neuro‐osseous constraints affecting trajectory selection.

A major finding of this study was the identification of a candidate balanced trajectory window (CPA 20°–25° and CSA 25°–30°). This angular range should not be interpreted as a universally optimal trajectory, but rather as a region achieving a favorable compromise between multiple competing anatomical factors. Specifically, increasing CSA effectively reduced iliac crest and S1 SAP obstruction, but excessive angulation could increase interaction with certain osseous structures. Conversely, increasing CPA reduced L5 nerve root intersection but was associated with increased interference with the L5 inferior articular process.

These findings highlight an inherent trade‐off in L5/S1 TELD planning: improving access through one anatomical constraint may increase the risk of violating another structure. Therefore, trajectory selection should not rely on a single anatomical parameter but require integrated consideration of multiple neuro‐osseous factors. The proposed trajectory window provides a quantitative reference for this balance rather than a definitive surgical prescription.

### 4.3 Implications for Surgical Planning

The quantitative framework developed in this study may provide a foundation for future computer‐assisted surgical planning systems. Current preoperative planning methods largely depend on surgeon experience, two‐dimensional imaging interpretation, or isolated anatomical measurements. By converting patient‐specific MRI anatomy into measurable spatial constraints, our approach provides a potential pathway toward more standardized and reproducible trajectory assessment.

Recent work by Ukeba et al. further illustrates the translational potential of AI‐assisted three‐dimensional imaging for endoscopic trajectory planning. In a retrospective series of 40 patients undergoing L5/S1 full‐endoscopic transforaminal discectomy, AI‐assisted 3D MRI/CT fusion imaging was used to establish a virtual endoscopic axis while accounting for the iliac crest, SAP, and exiting nerve root. The simulated axial and sagittal insertion angles showed close agreement with the actual postoperative endoscopic insertion angles, and favorable short‐term clinical outcomes were reported [26]. This study provides important evidence that patient‐specific three‐dimensional simulation can be translated into clinical trajectory planning when the simulated trajectory is directly compared with operative execution. In contrast, the present study addresses a preceding anatomical modeling question rather than validating a patient‐specific surgical plan. By systematically sampling a predefined trajectory space, we sought to characterize how iliac, neural, dural, and facet‐related constraints interact across different angular combinations. Accordingly, the candidate trajectory range identified in our study should be interpreted as an anatomically favorable range that provides quantitative boundary conditions for subsequent patient‐specific planning and clinical validation, rather than as a clinically validated surgical recommendation.

The clinical value of this framework lies not in replacing surgeon judgment, but in providing objective anatomical information that may assist decision‐making. Future navigation or robotic systems could potentially integrate these quantitative constraints with intraoperative imaging registration to dynamically adjust planned trajectories according to patient‐specific anatomy and operative conditions.

However, the current study was designed to characterize anatomical feasibility rather than predict the exact puncture trajectory selected by surgeons. Actual intraoperative trajectories are influenced by multiple factors, including surgeon experience, fluoroscopic feedback, and technical adjustments. Therefore, future studies incorporating standardized intraoperative trajectory acquisition and clinical outcome assessment will be required to further evaluate the translational potential of this framework.

### 4.4 Strengths and Limitations

The major strengths of this study include the integration of high‐resolution 3D T2W‐SPACE MRI, AI‐assisted automated reconstruction, and systematic simulation of 2940 virtual TELD trajectories. Unlike conventional CT‐based approaches focusing primarily on osseous anatomy, our framework simultaneously incorporates neural and bony structures within a unified three‐dimensional environment, enabling quantitative analysis of the competing constraints influencing L5/S1 TELD.

Several limitations should be acknowledged. First, the reconstructed models were generated from preoperative MRI acquired in the supine position, which may differ from the intraoperative prone position due to changes in lumbar alignment. Nevertheless, preoperative imaging remains the basis of current navigation workflows, and the proposed framework provides anatomical constraints that can be further adjusted during intraoperative registration.

Second, the identified trajectory window was derived from geometric simulation and was not directly correlated with intraoperative trajectories or postoperative outcomes. Importantly, the aim of this study was to characterize anatomical constraints governing TELD trajectory selection rather than to predict surgeons' actual puncture trajectories, which may be influenced by individual preference and intraoperative adjustments. Future prospective studies incorporating standardized trajectory acquisition and clinical validation are warranted.

Third, although high‐resolution MRI and AI‐based segmentation enabled patient‐specific reconstruction, inherent limitations related to image resolution, segmentation accuracy, and model approximation may influence simulated trajectory measurements. In addition, validated clinical thresholds for acceptable intersection volumes are currently unavailable, and the present framework does not incorporate a formal weighting function among different anatomical constraints. Therefore, the identified trajectory range should be interpreted as a candidate balanced trajectory window rather than a definitive automated surgical plan.

## 5 Conclusion

In conclusion, this study provides a quantitative 3D analysis of the L5/S1 surgical corridor for TELD using an automated segmentation pipeline followed by rule‐based geometric trajectory simulation. The findings suggest that a CPA of 20°–25° combined with a CSA of 25°–30° represents a candidate balanced trajectory zone. This geometric window may help navigate the anatomical constraints of the lumbosacral junction by reducing iliac crest obstruction while considering the L5 nerve root, dural sac, and articular processes. These quantitative geometric constraints may provide a preliminary framework for future computer‐assisted trajectory assessment systems.

## Author Contributions

**Yunfei Wang:** conceptualization, methodology, software, data curation, writing – original draft, writing – review and editing, funding acquisition, formal analysis. **Chengjie Huang:** data curation, methodology, software, writing – original draft. **Qingqing He:** conceptualization, methodology, data curation. **Junjie Lu:** data curation, validation, software, visualization. **Mengdi Li:** software, methodology, visualization. **Xiang Liu:** data curation, software, methodology. **Jingcheng Zhou:** data curation, investigation. **Ce Bian:** software, methodology, visualization. **Guiqian Zhang:** data curation, investigation. **Haotian Luo:** validation, visualization, software, data curation. **Rui Zhang:** conceptualization, data curation. **Feng Liu:** conceptualization, data curation. **Xionggang Yang:** project administration, resources, conceptualization, methodology. **Zhihai Su:** software, methodology, conceptualization, project administration, supervision. **Sheng Lu:** writing – review and editing, conceptualization, project administration, methodology, funding acquisition, supervision.

## Funding

This study has received funding by the National Natural Science Foundation of China (82560436, 82660524, and 82604004); Yunnan Key Laboratory of Digital Orthopaedics (202005AG070004); Yunnan Clinical Medical Center for Spinal Cord Diseases (ZX2022000101); “Yun‐Ling Scholars” Program of Yunnan Province (XDYC‐YLXZ‐2022‐0015); Yunnan International Joint Laboratory of Intelligent Orthopaedics (202503AP140037); Postdoctoral Workstation Project of Yunnan Provincial First People's Hospital (KHBSH‐2025‐09); and Young Science and Technology Talent Support Program of Guangdong Precision Medicine Application Association; Youth S&T Talent Support Programme of Guangdong Provincial Association for Science and Technology (SKXRC2026141). The Yunnan Provincial Caiyun Postdoctoral Innovation Project (KH‐CYBSH‐2026‐01).

## Ethics Statement

This study was approved by the Ethics Committee of The Fifth Affiliated Hospital of Sun Yat‐sen University (Approval No: 2020 K05‐1). As the retrospective data modeling component of a prospective study that has been registered with the Chinese Clinical Trial Registry (ChiCTR2100052883), all patients signed informed consent forms prior to the commencement of the prospective study. The study was conducted in accordance with the Strengthening the Reporting of Cohort Studies in Surgery Guidelines (STROCSS 2021) and the latest version of the Declaration of Helsinki.

## Conflicts of Interest

The authors declare no conflicts of interest.

## Supporting information


**Table S1:** Results of the intersection volumes between the virtual working channel with the L5 atypical structures, L5 all structures, dural sac, S1 atypical structures, and S1 all structures from different angles.

**Video S1:** Continuous recording of the MRI‐based segmentation and three‐dimensional reconstruction workflow. The video demonstrates the complete workflow from import of raw 3D T2W‐SPACE MRI DICOM data to deep learning‐based segmentation, generation of voxel‐based anatomical masks, and three‐dimensional reconstruction of lumbosacral structures. The reconstructed structures include the L5 and S1 vertebrae, L5/S1 intervertebral disc, ilium, L5 nerve roots, dural sac, and skin.

## Data Availability

The data that support the findings of this study are available from the corresponding author upon reasonable request.
