# Supplementary material for: Balancing Iliac Crest Avoidance and Neural Safety in L5/S1 TELD: Quantitative Identification of a Candidate Balanced Trajectory Window Using AI‐Assisted 3D MRI Reconstruction
Source: Orthop Surg. 2026 Nov 5:10.1111/os.70433. Online ahead of print. doi: 10.1111/os.70433 (PMC13738153; doi:10.1111/os.70433)
Supplement: Supplementary file 1 [file OS-9999-0-s002.docx]

Supplemental Table 1 Results of the intersection volumes between the virtual working channel with the L5 atypical structures, L5 all structures, dural sac, S1 atypical structures and S1 all structures from different angles.

| Different angle and intersection volumes | CPA 0° | CPA 5° | CPA 10° | CPA 15° | CPA 20° | CPA 25° | CPA 30° |
| --- | --- | --- | --- | --- | --- | --- | --- |
| CSA 0° |  |  |  |  |  |  |  |
| L5 R | 23.54 | 21.09 | 37.98 | 9.06 | 16.38 | 2.26 | 3.99 |
| L5 All | 23.74 | 21.92 | 39.20 | 16.19 | 44.54 | 101.35 | 183.26 |
| Dural sac | 21.71 | 34.10 | 44.52 | 65.94 | 72.66 | 93.78 | 120.08 |
| S1 R | 437.24 | 533.65 | 520.69 | 452.24 | 325.49 | 169.57 | 78.34 |
| S1 All | 546.94 | 754.64 | 832.70 | 761.18 | 599.64 | 394.70 | 257.53 |
| CSA 5° |  |  |  |  |  |  |  |
| L5 R | 46.77 | 23.69 | 31.24 | 15.38 | 4.21 | 4.34 | 6.93 |
| L5 All | 46.07 | 24.39 | 31.70 | 25.98 | 59.82 | 133.96 | 218.64 |
| Dural sac | 26.07 | 39.45 | 52.22 | 65.86 | 82.84 | 104.72 | 126.31 |
| S1 R | 243.41 | 324.66 | 337.97 | 273.40 | 143.49 | 71.54 | 38.82 |
| S1 All | 306.62 | 474.80 | 529.08 | 505.80 | 422.00 | 316.92 | 196.24 |
| CSA 10° |  |  |  |  |  |  |  |
| L5 R | 130.52 | 76.57 | 43.42 | 21.40 | 21.58 | 23.61 | 13.68 |
| L5 All | 129.46 | 76.86 | 47.67 | 37.36 | 85.57 | 176.36 | 268.80 |
| Dural | 33.32 | 46.14 | 60.48 | 73.28 | 93.20 | 112.80 | 135.71 |
| S1 R | 102.10 | 144.13 | 133.70 | 95.06 | 49.97 | 24.14 | 4.74 |
| S1 All | 140.22 | 250.04 | 303.69 | 325.71 | 289.67 | 213.51 | 125.04 |
| CSA 15° |  |  |  |  |  |  |  |
| L5 R | 338.04 | 241.03 | 116.98 | 63.42 | 61.15 | 58.71 | 46.55 |
| L5 All | 336.17 | 238.91 | 119.81 | 75.48 | 119.59 | 202.03 | 292.01 |
| Dural sac | 31.01 | 42.30 | 63.44 | 79.79 | 99.11 | 123.06 | 151.65 |
| S1 R | 32.92 | 40.03 | 39.54 | 22.39 | 12.63 | 5.17 | 1.29 |
| S1 All | 61.47 | 106.32 | 172.97 | 181.45 | 176.61 | 127.41 | 77.78 |
| CSA 20° |  |  |  |  |  |  |  |
| L5 R | 618.86 | 504.76 | 281.56 | 141.71 | 123.19 | 136.95 | 116.44 |
| L5 All | 615.44 | 495.33 | 278.45 | 154.80 | 168.57 | 239.81 | 309.16 |
| Dural sac | 40.20 | 53.04 | 69.93 | 90.29 | 114.06 | 140.84 | 170.08 |
| S1 R | 5.42 | 5.10 | 5.13 | 4.53 | 2.65 | 10.90 | 0.11 |
| S1 All | 22.85 | 46.76 | 76.80 | 97.06 | 91.92 | 78.73 | 46.26 |
| CSA 25° |  |  |  |  |  |  |  |
| L5 R | 850.17 | 731.38 | 462.87 | 273.65 | 226.05 | 201.42 | 217.66 |
| L5 All | 842.47 | 714.83 | 454.00 | 283.85 | 249.69 | 273.90 | 308.50 |
| Dural sac | 51.48 | 66.55 | 85.24 | 107.75 | 133.44 | 161.57 | 191.37 |
| S1 R | 1.40 | 0.80 | 0.41 | 0.36 | 0.23 | 2.87 | 0.04 |
| S1 All | 11.88 | 22.34 | 35.29 | 43.86 | 43.27 | 38.65 | 27.23 |
| CSA 30° |  |  |  |  |  |  |  |
| L5 R | 911.54 | 822.80 | 599.72 | 428.04 | 349.80 | 289.61 | 246.33 |
| L5 All | 908.35 | 811.65 | 594.78 | 435.93 | 358.58 | 327.40 | 316.81 |
| Dural sac | 64.87 | 82.31 | 103.48 | 128.68 | 156.53 | 185.96 | 207.67 |
| S1 R | 0.42 | 0.09 | 0.07 | 0.92 | 0.06 | 0.02 | 0.01 |
| S1 All | 6.66 | 9.91 | 15.14 | 20.09 | 44.39 | 18.51 | 16.82 |

Note: Data are means of intersection volume.

Abbreviations: CPA, coronal plane angle; CSA, cross section angle; L5 R, the atypical structures of the 5th lumbar vertebra bone (including the vertebral body, pedicles, and transverse processes); L5 All, all structures of the 5th lumbar vertebra bone; S1 R, the atypical structures of the 1st sacral vertebra bone (including the vertebral body, pedicles, and transverse processes); S1 All, all structures of the 1st sacral vertebra bone.
